# Effect of Clove (*Syzygium aromaticum*) spice as microbial inhibitor of resistant bacteria and Organoleptic Quality of meat

**DOI:** 10.1016/j.sjbs.2021.03.052

**Published:** 2021-03-27

**Authors:** Rebecca Tshabalala, Adia Kabelinde, Christ-DONALD KAPTCHOUANG TCHATCHOUANG, Collins Njie Ateba, Madira Coutlyne Manganyi

**Affiliations:** a Department of Microbiology, North West University – Mafikeng Campus, Private Bag X2046, Mmabatho 2735, South Africa; b Food Security and Safety Niche Area, Faculty of Agriculture, Science and Technology, North-West University, Mafikeng Campus, Private Bag X2046, Mmabatho 2735, South Africa; c Department of Biological and Environmental Sciences, Faculty of Natural Sciences, Walter Sisulu University PBX1, Mthatha 5117, South Africa

**Keywords:** Antimicrobial activity, Inhibition, Bacterial pathogens, Organoleptic, Meat, Spices

## Abstract

For centuries, spices have been utilized as flavourants, colourants and as preservatives in food. Moreover, spices possess various antimicrobial properties with massive health benefits for the treatment and management of ailments and diseases. The present study was focused on three (3) aspects; (1) isolation and molecular identification of bacteria from the meat; (2) to determine the antimicrobial activity of the spices against the pathogens; (3) to assess the organoleptic properties of the spiced meat. A total of twelve (n = 12) spices evaluated against forty (n = 40) spoilage food-borne pathogenic bacteria (*Escherichia coli* and *Enterococci* spp.). The spice extracts were tested using disk diffusion method to determine the inhibition abilities. The results show that clove and black seed cumin extract exhibited excellent antibacterial activity against most pathogenic bacteria. Clove displayed the highest inhibition zone of 18 mm against *E. coli (*EcFwS1)*.* Clove extract was the most inhibitor followed by black cumin, whereas extracts of thyme and cinnamon showed weak antibacterial activities against the tested strains. The most sensitive strain to spice extracts was *Enterococcus* spp. (EnFmL1) and the most resistant strain being *E. coli.* (EcFmS1 and EcFpL1). Untreated meat showed that *E. coli* and *Enterococcu*s spp. count was 4.4 * 10^5^ ± 3.4 * 10^5^ and 2.2 * 10^5^ ± 3.6 * 10^4^ cfu/mL respectively after 7 days while the single dose of clove showed 5.4 * 10^4^ ± 4.4 * 10^2^ cfu/mL of *E. coli* and 1.7 * 10^5^ ± 4.1 * 10^4^ cfu/mL of *Enterococcu*s spp. The organoleptic characteristics such as colour, texture, odour, pH, shape of the single dose of clove on the meat was overall acceptable.

## 1 Introduction

Food-borne disease resulting from consumption of meat contaminated with pathogenic bacteria is currently one of the leading causes of morbidity. Cross contamination occurring within the premises, as well as no proper time–temperature control for heating, cooling and storage are considered as a main cause of contamination (Al-Kutby, 2012). Poor hygienic practices by personnel in the meat industry occurs frequently therefore precautions such as preservation of perishable foods such as meat is imperative. Perishable products have shelf life of a week or a month, respectively in the presence of high doses of chemical preservatives (Dhillon, 2011). The use of chemical preservation is becoming a major concern to human health. Therefore natural preservatives are coming up as an option. The International Standard Organization (ISO) define spices as “*vegetable products or mixtures thereof, used for flavouring, seasoning, and imparting aroma in foods*”. There has been a growing interest in natural compounds extracted from herbs and spices (Srinivasan, 2014) and derived extracts have been utilized since ancient times to improve sensory characteristics of food, as preservatives, for their nutritional and healthy properties and also as their antimicrobial effects (Martínez-Graciá et al., 2015). According to Monte et al. (2014); spices and herbs are rich sources of phytochemicals, bioactive compounds derived from plants that are capable to inhibit bacterial growth by damaging microbial membrane structures. These bioactive compounds consist of vitamins C, E, carotenoids and phenolic compounds, flavonoids, tannins, and flavones that are known for their beneficial role (Sokamte et al., 2019). The biological roles include the inhibition of oxidative stress, anti-inflammation, anti-hypertension, anti-microbial and inhibition of cardiovascular disease (Li et al., 2019) amongst others. Different types of spices exhibit different antimicrobial activity depending upon the nature of the spice whether dry, fresh or extracted forms (Saeed et al., 2016). For centuries, nutritional value content in red meat made it a valuable food source for humankind (Pal et al., 2018). Wyness (2016) stated that “Red meat has been an important part of the human diet throughout human evolution; it provides a rich source of high biological value protein and essential nutrients, some of which are more bioavailable than in alternative food sources”. There is a massive zoonosis disease to consumers if not produced and handled properly (Nisar et al., 2018) during production (Akindolire et al., 2015).

Raw meat has a water activity of 0.98–0.99, pH ranging from 5.5 to 6.5 and this is a satisfying requirement for microbial growth conditions (Ateba and Mochaiwa, 2014). Ateba and Mochaiwa (2014) further mention that the presence of these invisible microbes in meat products increases the chance of contracting foodborne illnesses. Liu et al. (2017) stated that microbial pathogens are the major contributors of food spoilage which leads to foodborne illnesses, emergence of multidrug resistant bacteria such as *Staphylococcus aureus* (*S. aureus*), *Escherichia coli* (*E. coli*), *Enterococcus* species such as (*E. faecium* and *E. faecalis*), and other microbial pathogens (Liu et al., 2017). These pathogens are also associated with diarrheal diseases. The meat industry is enormous in South Africa; thus multiple outbreaks and previous studies have disclosed that some personnel in food industries often lack basic food safety knowledge when it comes to keeping the right temperature for storage, personal hygiene and the prevention of cross-contamination during slaughtering (Sibanyoni et al., 2017), transportation and handling in the retail market.

Microbial pathogens leads to food-borne illnesses. Foodborne illnesses are a major healthcare concern worldwide. Global burden of disease (GBD) in 2015 estimated that foodborne diarrheal diseases as the sixth leading cause of global disability-adjusted life years (DALYs) (Keisam et al., 2019). Bisholo et al. (2018) estimated that 92 million people fall ill from consuming contaminated foods especially meat and meat products, resulting in 137 000 deaths annually in Africa, however it is difficult to estimate the burden of food-borne disease mortality rate in South Africa due to lack of data (Bisholo et al., 2018). The retail market and scientists are working tirelessly to reduce contamination in food products especially those of animal origin by applying measures such as radiation and inorganic chemicals. Nevertheless, it is considered that chemical synthetic preservatives have many carcinogenic and teratogenic consequences as well as residual toxicity (Costa et al., 2019). Since these measures tend to be detrimental to human lives, the industry shift is pointing towards natural forms. According to Taguri et al., (2004) studies have revealed that, in addition to their inhibitory effects on exotoxins, polyphenols of various foods and herbs are beneficial to human health, and some extracts of polyphenol-rich plants (herbal spices) have been applied to functional foods or supplements. The current study will be focusing on using herbal spices as antimicrobial agents against food-borne pathogens. The aim of this study was to determine antimicrobial activity of spices against food-borne pathogens isolated from raw meat.

## 2 Materials and methods

### 2.1 Collection of meat samples

A total of twelve (n = 12) meat samples (raw steak, raw mutton, raw mince and raw pork) were collected from three different retail supermarkets in North-West Province, South Africa. Four (N = 4) samples were collected from each supermarket in the area. Approximately 100g of meat samples were purchased and transported to North-West University – Mafikeng, Molecular Microbiology laboratory for microbiological analysis.

### 2.2 Isolation of bacterial strains from meat sample

The target microbes in this study were Escherichia coli, Staphylococcus aureus and *Enterococcus* species and the samples were processed within 48 h of collection. Samples were cut aseptically into thin smaller pieces of 1 g using sterile scalpel. One (1) gram of meat samples were dissolved in 9 mL peptone water and homogenized by vortexing for 1 min. All samples were subjected to a 10-fold serial dilution (10-1-10-6. From the 10-4-10-6 tubes an aliquot (100 μL) of the diluent was spread-platted onto Nutrient agar (Merck, Germany) a general media. Each diluent was prepared in triplicates. The plates were incubated aerobically at 37 °C for 18–24 h. Total microorganism count was counted and expressed in Colony Forming Unit per 100 mL (Bantawa et al., 2018). The diluent from the10-4 tube was further plated on selective media. For the isolation of Escherichia coli the rinsate was plated onto Eosin Methylene Blue agar (EMBA, Merck, Germany), Staphylococcus aureus, the rinsate was plated onto Mannitol Salt agar (MSA, Biolab, South Africa), *Enterococcus* spp., the sample rinsate was plated onto Bile Esculin Agar (BEA, Merck, Germany). Samples were incubated aerobically at 37 °C for 24 h. Appearance of black colonies on Bile esculin agar was presumptively identified as enterococci and green metallic sheen on Eosin Methylene Blue agar as *E. coli,* a single colony from each medium plate was sub-cultured for purification. Presumptive positive colonies were subjected to biochemical identification tests and Gram straining (Mulamattathil et al., 2014). Colonies were purified by sub-culturing a single colony using the streaking plate method on different selective media and incubated aerobically at 37 °C for 24 h.

### 2.3 Bacterial identification tests

#### 2.3.1 Gram-staining

Isolates were Gram-stained using standard techniques (Cruickshank, 1975), which differentiates bacterial species into Gram-positive and Gram-negative based on the chemical and physical properties of their cell walls. Colonies were retained for further identification (Akindolire et al., 2015).

#### 2.3.2 Preliminary biochemical identification of isolates

Biochemical characterisation of the bacteria were conducted by performing specific tests such as catalase, oxidase, TSI, Esculin hydrolysis and 6.5% NaCl.

### 2.4 Molecular identification

#### 2.4.1 Genomic DNA extraction

Deoxyribonucleic acid (DNA) extraction was performed using the boiling method as described by Maugeri et al. (2004). Fresh pure colonies were suspended in nutrient broth and incubated at 37 °C for 24 hrs. After incubation, samples were transferred into 1.5 mL Eppendorf tubes and were vortexed and further transferred into centrifuge tubes and centrifuged (Tomos, Singapore) at 15,000*g* for 15 min. The supernatant was eliminated and the pellet was re-suspended in molecular biology-grade water (Eppendorf, Hamburg, Germany) and centrifuged at 15,000*g* for 10 min. The supernatant was eliminated and the pellet was re-suspended in 200 μL of sterile nuclease-free water, subjected to boiling at 100 °C in a heating block for 10–15 min using MS2 a Dri-Block DB.2A (Tech, South Africa) and centrifuged at 13,500*g* for 5 min. before storing at −20 °C. Aliquots (5 μL) of template DNA was used for PCR amplification.

#### 2.4.2 Agarose gel electrophoresis of DNA extraction

The presence of DNA extracts was confirmed by electrophoresis on a 1% (w/v) agarose gel in 1X TAE buffer. The gel separation was done at 80 V for 45 min according to protocol and the electrophoretic pattern was analysed on a UV illuminator (Bio-Rad Laboratories, USA) in the Molecular Microbiology laboratory at the North-West University Mafikeng.

#### 2.4.3 PCR amplification of 16SrRNA gene fragments

Amplification of targeted genes was performed using Polymerase chain reaction (PCR) analysis. For the amplification, 1 μL of DNA was added to 12.5 μL of master mix (2X DreamTaq Green Buffer, dATP, dCTP, dGTP, and dTTP, 0.4 mM each, and 4 mM MgCl2) (Thermo Scientific, USA), 0.5 μL (0.2 μM) of respective oligonucleotide primers and the reaction volume were made up with 11 μL nuclease free water. The final volume of 25 μL, PCR was performed in a thermal cycler (Bio-Rad Laboratories, USA). The amplification cycles consisted of an initial DNA denaturation at 95 °C for 1 min, followed by 25 cycles of denaturation at 94 °C for 1 min, primer annealing at 55 °C, for 1 min, extension at 68 °C for 2 min, and a final single elongation at 72 °C for 10 min.

#### 2.4.4 Identification of *E. coli* by PCR analysis

The identities of a total 20 presumptive *E. coli* isolates were determined through amplification of the uidA gene fragments (Anbazhagan et al., 2011). Amplifications were performed using DNA thermal cycler (model- Bio-Rad C1000 Touch TM Thermal Cycler). The PCR reactions were performed using the oligonucleotide primer sequences in Table 1. The reactions were prepared in 25 μL volumes that constituted 12.5 μL of 2X DreamTag Green Master Mix, 11 μL RNase free distilled water, 0.5 μL mixture of both the forward and reverse primers and 1 μL of template DNA. The PCR reagents were obtained from the Inqaba Biotechnical Industry Ltd, Sunnyside, Pretoria, South Africa. PCR conditions for *E. coli*: 1 cycle of 10 min at 95 °C, 35 cycles of 45 s at 95 °C, 30 s at 59 °C, 1 min 30 s at 72 °C; 1 cycle of 10 min at 72 °C.

**Table 1:** Oligonucleotide primers used for amplification.

Species/target gene	Primers Sequence (5′ – 3′)		Amplicon Size	Ref.
*E. coli* 16S rRNA	Forward 27F	AGAGTTTGATCATGGCTCAG	1420 bp	Korzeniewska and Harnez, 2013
	Reverse 1492R	GGTACCTTGTTACGACTT		
UidAF	Forward	AAA ACG GCA AGA AAA AGC AG	147 bp	Anbazhagan et al., 2011
UidAR	Reverse	ACG CGT GGT TAA CAG TCT TGC G		
*Enterococci* 16S rRNA	Forward	TGCATTAGCTAGTTGGTG	356 bp	Kariyama et al., 2000
	Reverse	TTAAGAAACCGCCTGCGC		
*Enterococci* ddl	Forward	CACCTGAAGAAACAGGC	475 bp	
		ATGGCTACTTCAATTTCACG		

#### 2.4.5 PCR analysis for specific identification of *Enterococcus* spp

Species specific identification of *Enterococcus* spp was performed using specific primers that are shown in Table 1 (Jackson et al., 2004). Amplifications were performed using DNA thermal cycler (model- Bio-Rad C1000 Touch TM Thermal Cycler). The PCR reactions were performed using the oligonucleotide primer sequences in Table 1. The cycling conditions utilised were as follows: initial denaturation of 95 °C for 4 min and 30 cycles of 94 °C for I minute, 55 °C for I minute and 72 °C for I minute. A final elongation step was performed at 72 °C for I0 minutes and samples were held at 4 °C.

#### 2.4.6 Agarose gel of PCR amplicons

The positive PCR amplicons were displayed on the electrophoresis with a 1% (w/v) agarose gels (Sambrook et al., 1989). Gels were stained with ethidium bromide (1.0 μL) and electrophoresis was conducted using a horizontal Pharmacia biotech equipment system containing 1X TAE buffer for an hour at 70 V. A 1000 kb DNA molecular weight Ladder (#N0467S) obtained from New England Biolabs Ltd (UK) was included in each gel in order to confirm the sizes of the amplicons. Gels were viewed under the UV light Trans illuminator (Lasec, South and Africa) (Sambrook et al., 1989), and images were captured using a Bio-Rad imaging system (Model Bio-RAD ChemiDocTM MP Imaging System, UK) using Gene Snap software (version 6.00.22).

### 2.5 Preparation of spice extracts

#### 2.5.1 Extraction method of spices

Approximately 10 g of spices were grinded with mortar, pestle, and were dissolved in 90 mL of sterile distilled water to make 100 mL of aqueous extract (11%w/v). The mixture was placed at room temperature for 24 h in sterile flasks and were filtered through sterilized Whatman no.1 filter paper (Lasec, South Africa). After filtration, the extracts were evaporated in water bath (Monitoring and control Laboratories, Lyndhurst, South Africa) until a final volume of 50 mL extract was reached (Upadhyaya et al., 2018).

### 2.6 Antimicrobial sensitivity testing for the isolates

Antimicrobial sensitivity testing was performed using the Kirby Bauer’s disc diffusion method. Nutrient broth was prepared and pure colonies from nutrient agar were inoculated into it and the suspension was incubated overnight, an aliquot (100 μL) of the bacterial suspension was spread-plated and a lawn was performed on the surface of Mueller-Hinton agar (MHA, Biolab, Wadeville, South Africa). Diffusion discs were impregnated with spice extracts and were performed in triplicates on the surface of inoculated plates. The plates were incubated at 37 °C for 16–24 h. After incubation, the zone diameter was observed and measured using a ruler. Thereby the zone of inhibition was interpreted as susceptible (S), Intermediate (I) or resistant (R).

### 2.7 Organoleptic assay

Organoleptic properties of the meat samples were evaluated in the laboratory of Molecular Microbiology in the North-West University, Mafikeng.

#### 2.7.1 Preparation and evaluation of meat samples

Approximately 1 kg of beef steak was obtained from a local supermarket and transported to the laboratory. Upon arrival the meat was cut into six (6) equal pieces and two (2) pieces were untreated; one piece was used for bacterial load count, the other served as a control. The remaining four (4) pieces were treated with two (2) different spices at varying masses. Five (5) samples were refrigerated for seven (7) days at 4 °C. Results were observed on day one (1), day three (3) and day seven (7). A six-point hedonic scale was used, where six was extremely desirable while one was extremely undesirable (Siham, 2015).

#### 2.7.2 Bacterial load count

One (1) gram from the untreated sample was subjected to a 10-fold serial dilution (10-1-10-3). From the 10-1-10-3 tubes an aliquot (100 μL) was plated on three (3) different media, the Plate Count Agar (PCA) (Merck, Germany), Eosin Methylene Blue Agar (EMBA) for the presence of *E. coli* spp and *Enterococcus* spp. The plates were incubated aerobically at 37 °C for 18–24 hrs. Total microorganism count was measured and expressed in Colony Forming Unit per 100 mL (Bantawa et al., 2018). After the seventh (7th) days; microbial counts were repeated on both treated and untreated samples. Samples were incubated aerobically at 37 °C for 24 hrs and the bacterial load was recorded.

## 3 Results and discussions

### 3.1 Isolation of bacteria from meat sample

A total of twelve (n = 12) meat samples consist of mincemeat, pork, wors and steak were collected from three (n = 3) different supermarkets in the area of Mmabatho North-West Province and screened for bacterial load, the presence and the prevalence of *Escherichia coli, Enterococcus* species and *Staphylococcus aureus* in Table 1; shows the results obtained. A total viable count and prevalence was reported under Section 4.2.

### 3.2 Determination of bacterial load and the prevalence of pathogens in the meat samples

The results shown in Table 1, indicates that mincemeat from shop Sm had the highest mean value of 300 cfu/mL as compared to other supermarkets, followed by Wors at 293 colonies counted from shop Sw. The steak from shop Ps had 46 colonies and followed by pork from shop Pp at a value of 83 cfu/mL, which was the least number of colonies isolated. A total of twelve (n = 12) meat samples comprising of mincemeat, pork, wors and steak were collected form three (n = 3) different supermarkets in the area of Mmabatho North-West Province and screened for bacterial load, the presence and the prevalence of *Escherichia coli, Enterococcus species* and *Staphylococcus aureus* in Table 1; shows the results obtained. These include a mean total viable count of meat samples that were collected from these various supermarkets and the number in percentages (%) for the prevalence of *E. coli and Enterococci* species. However, no *S. aureus* was detected in any of the samples.

TVC = Total viable count; TCC = Total coliform count

### 3.3 Determination of prevalence of Escherichia coli in the meat samples

**Fig.1:**
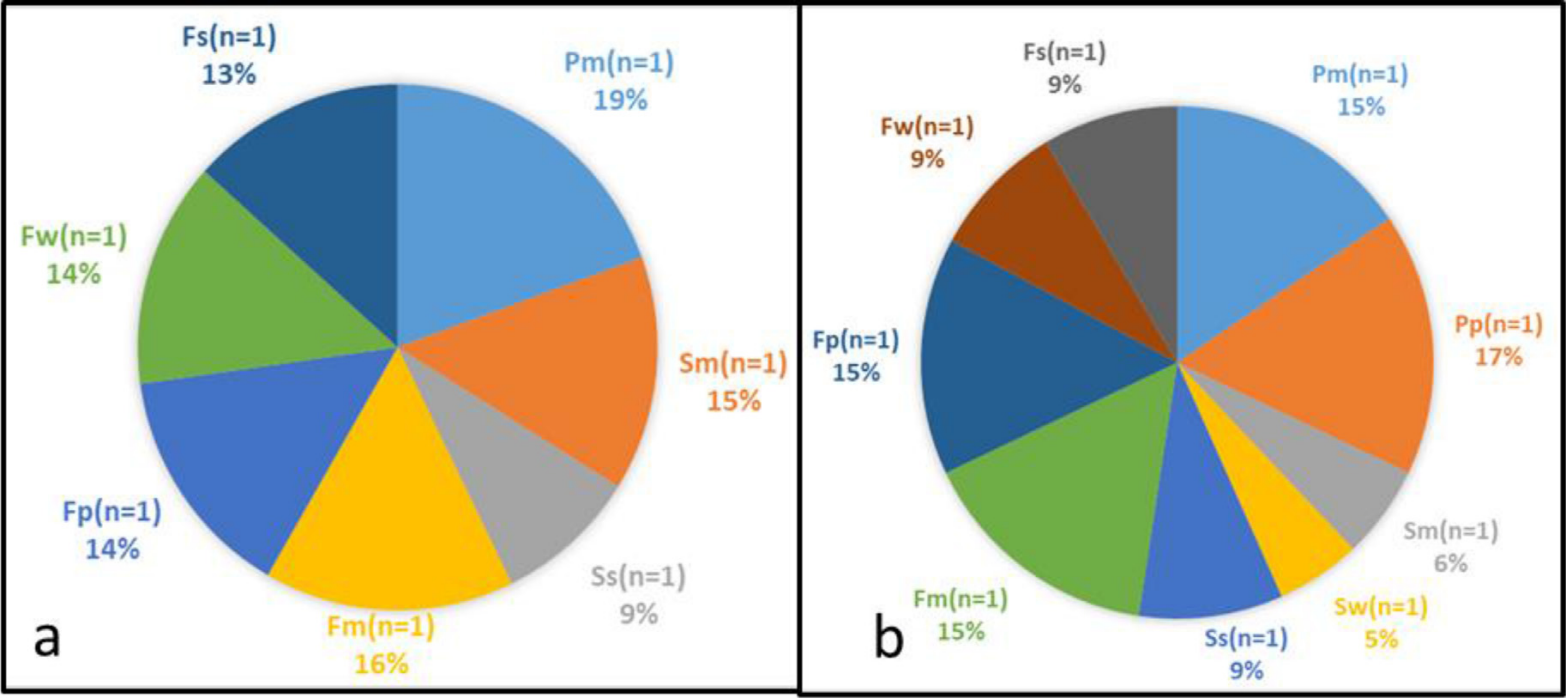
(a) Mean Total Viable Count for *E. coli* (n = %) (b) Mean Total Viable Count for *Enterococcus* sp. (n = %).

The overall prevalence of *E. coli* was 60% (186.1/333) of the total samples. At this level, considering hygiene and sanitary quality, the presence of *E. coli* indicate that consumers are at risk of exposure to a food-borne disease. This prevalence of *E. coli* in meat samples from various supermarkets in Mmabatho compare to other studies was, lower than that showed by previous study of Bantawa et al., 2018, yet higher than that of (Al-Mutairi, 2011) sampled from various supermarkets in Giza governorate, Egypt. The meat sample that showed the highest contamination by *E. coli* was pork from Pp shop at 17% and followed by pork from shop Fp, mincemeat from shop Pm and Fm all at 15%. The shop that indicated lower contamination by *E. coli* was Shop Sw at only 5% documented in Fig. 1a.

### 3.4 Determination of prevalence of *Enterococcus* spp. in meat samples

Fig. 1b indicates the overall prevalence of *Enterococcus* spp as 51% (179.8/353) for all the samples as compare to other studies which was much lower than those reported by Hayes et al., 2003, Mcgowan et al., 2006, Koluman et al., 2009 who found, respectively, 99%, 73.5% and 80% of raw meat contaminated by enterococci. Nineteen (19%) of the microorganism was isolated from the mincemeat in shop Pm which at this stage was the highest contaminated and the least being the steak from shop Ss at 9%.

### 3.5 Presumptive identification of isolates

Pure isolation was achieved with Bile esculin agar and Eosin methylene blue agar that facilitates growth of organisms belonging to the genus spp and *E. coli,* respectively. *Enterococcus* species were presumptively identified by the presence of black colonies that hydrolysed the esculin incorporated into the media and *E. coli* identified by a green metallic sheen colour which indicate lactose fermentation, although most of the colonies were identified as pink/lavender. According to literature, pink/ lavender colonies on Eosin Methylene Blue Agar indicate non-lactose fermenters. With their ability to hydrolze esculin in the presence of bile, enterococci possess a morphological appearance of cocci that grow in chains or in singles following Gram staining. Fourteen (14) of the twenty (20) isolates satisfied the morphological characteristics of a Gram positive cocci for enterococci (Montwendi, 2013).

#### 3.5.1 Biochemical assay

In TSI stabbing, the suspected *E. coli* of the 20 samples, 8 showed yellow butt (acid) and red slant (alkaline) with gas production and only three showed yellow butt and yellow slant without gas production. None of the isolates showed any blackening therefore depicting no production of hydrogen sulphide. All the 20 Gram positive cocci and 20 Gram negative rods were subjected to the catalase test and results are shown in Table 2. A small proportion 15 (75%) of the Gram positive isolates grown on BEA were able to breakdown hydrogen peroxide despite the fact that a few catalase negative strains were observed. This observation suggests that some *E. faecium* isolates are usually catalase negative whereas E. faecalis are frequently catalase positive (Montwendi, 2013) as most literature would point out. All suspected *E. coli* isolates tested positive. The suspected Enterococci spp were tested for their ability to grow on 6.5% NaCl, majority of the isolates satisfied the finding. About 10% proved otherwise, therefore the results prove that the microorganisms could be Enterococci and for the fact that they did hydrolyse bile on BEA. The identities of presumptive *E. coli* isolates were also tested for the production of cytochrome *c* oxidase enzyme. A large portion of the isolates proved to belong to the genus and spp.

**Table 2:** Determination of antimicrobial activity of spice water extract against pathogens by disc diffusion method.

Zone of inhibition: − no activity; + slight activity (>5mm); ++ good activity (6–10 mm); +++ very good activity (>10 mm).

### 3.6 Molecular identification

#### 3.6.1 Extraction of DNA from presumptive *E. coli* isolates

DNA was extracted from all presumptive *E. coli* and Enterococci spp. isolates. DNA extraction was successful in the study, which was confirmed by electrophoresis on a 1% (w/v) agarose gel and DNA was of high quality.

#### 3.6.2 The 16SrRNA gene analysis of presumptive *E. coli* and *Enterococci* spp.

Bacterial 16S rRNA gene fragments were amplified from all the 20 presumptive isolates of *E. coli* and 20 presumptive isolates for Enterococci spp. All the isolates were positive for the universal 16S rRNA gene PCR analysis. A 1% (w/v) agarose gel image of bacterial 16S rRNA gene fragments amplified during the study. PCR amplicons possessed the expected size (1420 bp) and all these isolates were subjected to an *E. coli* and Enterococci species-specific PCR assay.

#### 3.6.3 Proportion of isolates confirmed as *E. coli* through PCR amplification of uidA gene

Escherichia coli species specific PCR was performed on all isolates that were positive for 16S rRNA gene by amplifying the uidA *E. coli* housekeeping gene. Isolates containing the uidA gene fragment and were therefore confirmed as *E. coli* isolates. The expected amplicon size of 147 bp was obtained. Fig. 1c indicates a 1% (w/v) agarose gel image of uidA gene fragments amplified from *E. coli* isolates and the *E. coli* positive control strain.

#### 3.6.4 Proportion of isolates confirmed as *Enterococcus* spp. through PCR amplification of ddl gene

Enterococci spp. strains were confirmed using ddl gene. After running the 1% (w/v) agarose gel, the bands were measured at 475 bp.

### 3.7 Antimicrobial profile of isolates

#### 3.7.1 Antibacterial activity of spice extracts

**Fig. 2:**
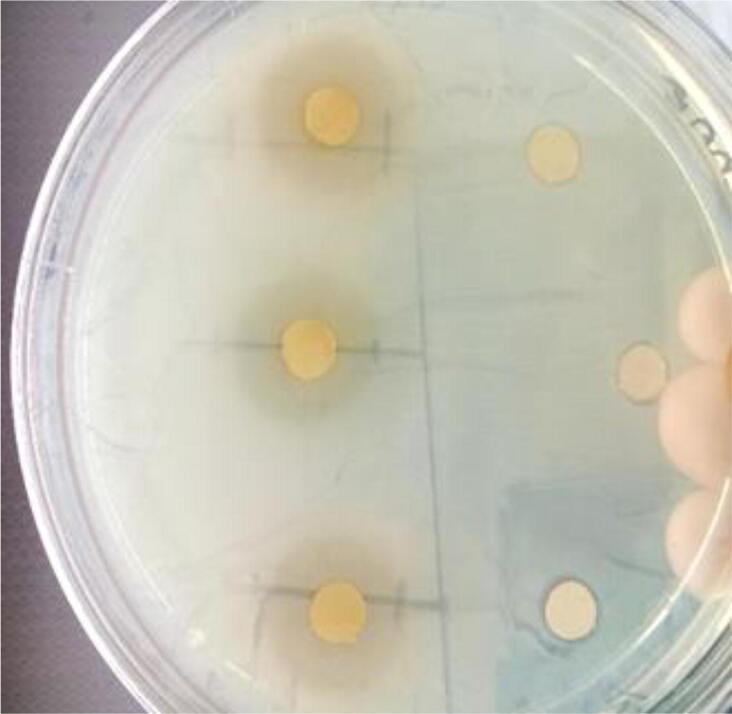
Effect of clove spice extract against Gram negative bacteria.

In the present study, the antimicrobial activities of twelve spice extracts were examined against food borne bacterial pathogens. Results obtained by agar well diffusion technique, as a qualitative method, were summarized in Table 3. The spice extracts were found to have potent antimicrobial activity against some of the Gram-positive and Gram-negative microorganisms tested. Clove represented in Fig. 2 and black cumin (*B. cumin*) extracts proved to be the most inhibitors against all tested bacteria. Results obtained for clove and cumin extract in the present study were found to be similar to those reported by Nanasombat and Lohasupthawee (2005), in their findings clove and cumin also exhibited (>10 mm) against the same pathogens tested in this study. According to Saeed et al. (2016) clove has vital role as a spice because of its high value for its therapeutic activity. The phenylpropene eugenol compound in clove is a well-known aromatic compound. Eugenol is the main necessary component of clove that has significantly higher antimicrobial properties against food micro-organisms. On the other hand, the spice extracts of cinnamon and thyme showed weak antibacterial activities against most of the tested samples. Our results correlates with Nzeako et al. (2006) in which they found thyme extract could only inhibit *S. aureus* and therefore concluded thyme to be an extract with limited or narrowed antimicrobial activity. The attribute the poor performance of spice water extracts to maybe evaporation during boiling. (Table 4).

**Table 3:** Bacterial load of meat sample before and after 4 °C of storage for over a period of 7 days.

Time / temp.	Source	Control mean value (cfu/mL)	*B. cumin* mean value (cfu/mL) Single Double dose		Clove mean value (cfu/mL) Single Double dose	
DAY 1 4 °C	Ec	1.3 * 10^2^ ± 6.5 * 10^1^	–	–	–	–
	Ent	2.3 * 10^2^ ± 5.4 * 10^1^	–	–	–	–

DAY 7 4 °C	Ec	4.4 * 10^5^ ± 3.4 * 10^5^	1.5 * 10^5^ ± 2.8 * 10^2^	1.1 * 10^5^ ± 2.3 * 10^2^	5.4 * 10^4^ ± 4.4 * 10^2^	9.1 * 10^4^ ± 2.1 * 10^2^
	Ent	2.2 * 10^5^ ± 3.6 * 10^4^	1.9 * 10^5^ ± 1.4 * 10^5^	1.0 * 10^5^ ± 1.5 * 10^4^	1.7 * 10^5^ ± 4.1 * 10^4^	8.9 * 10^4^ ± 2.1 * 10^2^

Ec = *E. coli*; Ent = *Enterococci* spp. Time / temp. Source Control mean value (cfu/mL) *B. cumin* mean value (cfu/mL) single/ double dose Clove mean value (cfu/mL) single/ double dose.

**Table 4:** The organoleptic test results for meat samples.

Storage temperature	Organoleptic parameters	Preservation time (days) with *B. cumin*				Preservation time (days) with Clove				Control preservation time (days)			
	**Days**	**0**	**1**	**3**	**7**	**0**	**1**	**3**	**7**	**0**	**1**	**3**	**7**
4 °C	Colour	6	6	4	3	6	6	4	3	6	6	5	2
	Texture	6	6	4	4	6	6	4	5	6	6	4	1
	Odour	6	6	5	5	6	6	6	6	6	6	4	1
	pH	3	3	3	4	3	3	3	4	3	3	4	5
	Shape	6	6	3	5	6	6	3	4	6	6	5	5
	Overall acceptability	6	6	6	5	6	6	6	5	6	6	4	1

Overall acceptability.

#### 3.7.2 Comparison of the sensitivity of bacterial strains to antimicrobial spice extracts

In the present study, isolates from different sample differed in their antimicrobial resistance patterns and similar observations have been reported. The results reflected great variation in the sensitivity of bacterial strains against spice extracts (Fig. 2), generally Gram-positive bacteria were found to be more sensitive than Gram-negative bacteria (Table 2). Most studies are in line with our results Al-Kutby (2012) *E. coli* (EcFmS1 and EcFpL1) was considered the most resistant bacterial strain being inhibited only by two of twelve spices (clove and *B. cumin*) and the least resistant being the *Enterococci* spp. (EnFmL1) inhibited by eight of twelve spices.

### 3.8 Organoleptic assay

#### 3.8.1 Bacterial load from the analysis

The bacterial load was determined at day 1 and on the final day (7th) while refrigeration at 4 °C (Table 3). The highest bacterial load observed was from the control sample on day seven (7) when compared to the bacterial load at the beginning (bacterial load increased). These numbers show that bacteria might climates to the environment, temperature in this instance and replicate with changes in pH being the main factor (Grāmatiņa et al., 2017). The lowest amount of bacterial load was recorded from the samples that were treated with a single dose of clove and black seed cumin spices. The findings of this study are in agreement with the work of Shadrack et al. (2012) in which they found rosemary spice to be effective at low doses, they attribute the effectiveness to bactericidal effects of rosemarinic acid.

#### 3.8.2 Evaluation of organoleptic parameters

**Fig. 3:**
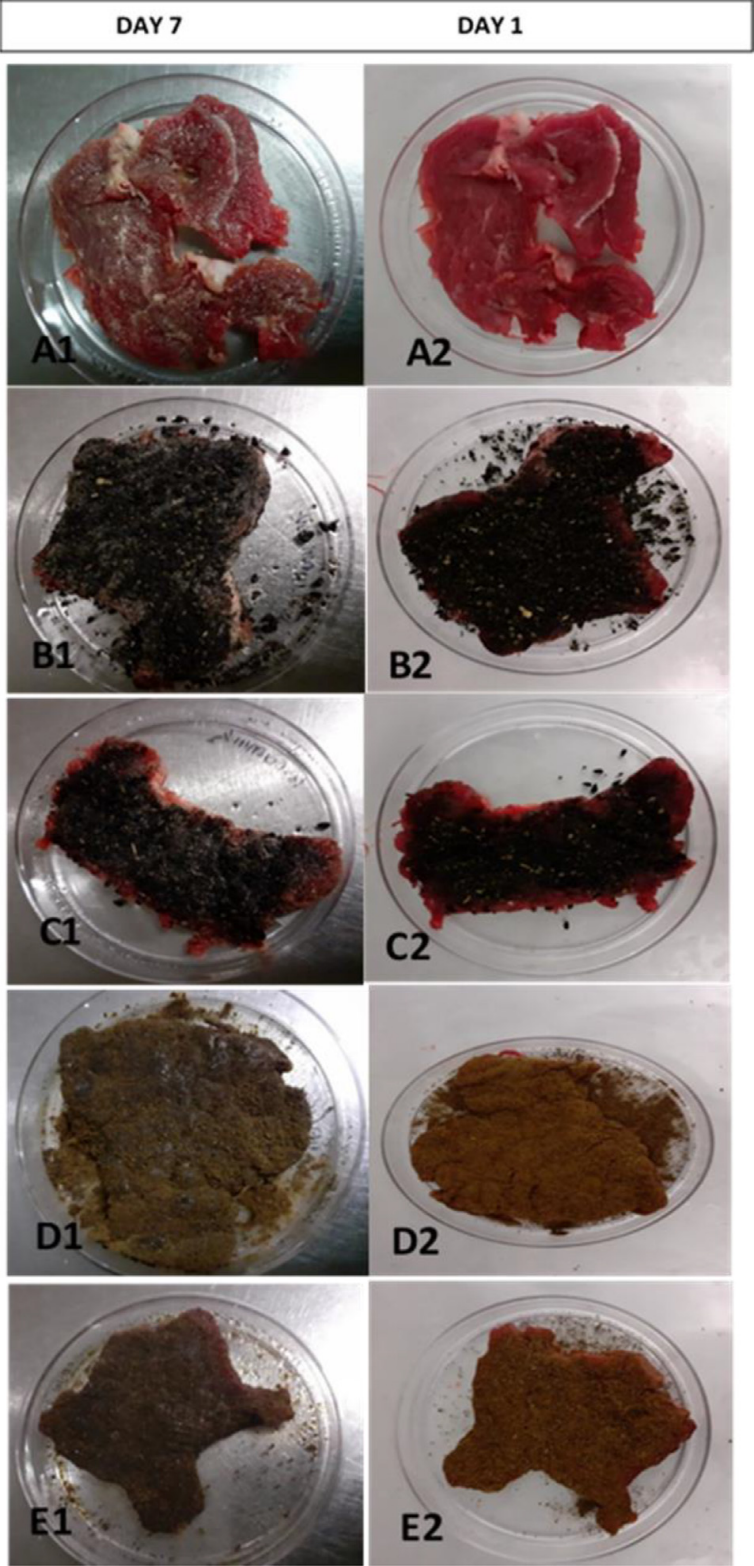
Meat samples on the first day (a) control, (b) meat treated with double the volume of black cumin, (c) meat sample treated with a single dose of black cumin, (d) meat sample treated with double the volume of clove, (e) meat sample treated with single dose of clove for day 1 and 7.

Meat samples were also evaluated based on the attributes such as colour, texture, odour, pH, shape and overall acceptability. Different levels of the attributes were chosen based on a 6-points hedonic scale from extremely bad (1) to extremely good (6) as shown in Table 5. Observations were done on the first and the last day of refrigeration. The freshness of meat samples change as proteins and fats start to break down, during storage. The morphological structure of muscle tissue also changes: meat secretes juice, the surface colour changes and unpleasant odour develops. Such meat is unfit for human consumption (Grāmatiņa et al., 2017). For the control sample, the first unpleasant changes in sensory parameters were detected on day 3 of storage, but for the meat samples with spice extract changes were observed on day 7 (the last day). Fig. 3 represents the overall appearance of the organoleptic assay on day 1 and 7.

**Table 5:** Organoleptic hedonic scale used to determine quality of meat Scale Colour Texture Odour pH Shape Overall acceptability.

Scale	Colour	Texture	Odour	pH	Shape	Overall acceptability
6	Extremely desirable	Extremely hard	Extremely pleasant	Extremely alkaline	Raised	Extremely acceptable
5	Very desirable	hard	Very pleasant	Alkaline	Slightly raised	acceptable
4	Moderately desirable	Moderately hard	Moderately pleasant	neutral	Flat	Slightly acceptable
3	Moderately undesirable	Moderately slimy	Moderately smelly	acidic	Slightly flat	Slightly unacceptable
2	Very undesirable	Very slimy	Very smell	Very acidic	Very flat	unacceptable
1	Extremely undesirable	Extremely slimy	Extremely smelly	Extremely acidic	Extremely flat	Discard

In terms of colour, all the samples (control and treated) from day 0 to day 3 appeared to be extremely desirable. However, on day 7, the treated samples were moderately undesirable while the untreated sample was very undesirable. The texture of the meat on day 1 to 3, all sample were soft and tender under cold storage. However, on the last day under the same conditions, treated samples appeared to be hard with the double dose being extremely hard. While the untreated sample was very slimy. The odour on day 0 to day 1, all meat sample had an extremely pleasant smell. And day 3, the smell began to change from being extremely pleasant to very pleasant for the samples treated with black cumin and moderately pleasant for the untreated sample. The sample treated with clove maintained the extremely pleasant smell. The pH from day 0 to 3, the pH of the meat samples was acidic and began changing to neutral for the treated samples. And to alkane to the untreated samples. Change in shape of the samples were observed from day 3 with the treated samples appearing to be slightly flat and the untreated samples begin slightly raised. The over-all acceptability on day 7 of refrigeration, treated meat samples were still acceptability and the unfit thus should be discarded.

## 4 Conclusion

As the world is aiming at a more sustainable and eco-friendly approaches, the use of natural preservatives have gained popularity because of its effectiveness and tremendous health benefits. Spices have been used since ancient times for combating food spoilage and contaminants, hence securing food safety. The findings in this study demonstrate that the meat in the retail supermarkets in Mafikeng, Mmabatho are highly contaminated with pathogenic bacteria*.* Clove and cumin have exhibited excellent antibacterial activity as compared to other spices such as rosemary, parsley, ginger, cayenne pepper and white pepper that were tested. Another advantage of using these herbs as antimicrobial agent is that, there is no harmful effect on body recorded so far and there is less chance of development of resistance in bacteria against these herbs (Shaheen et al., 2015). In case of the evaluation of organoleptic parameters, the treated meat samples yielded better results in term of the colour, odour, texture, shape, during the observation period compared to the control samples. Therefore, our data strongly supports the use of spices in reducing the pathogenic bacteria on meat and meat products. In conclusion, the results show the effect of herbal spices as natural preservatives to replace completely or partially the chemical preservatives (sodium nitrite etc). The use of chemical preservation is becoming a major concern to human health therefore natural preservatives are coming up as an option. In additionally, this study and other similar studies endorse the usage of spices as natural preservatives with antimicrobial properties in meat and establish their organoleptic effectiveness.

## Declaration of Competing Interest

All authors declare that there is no conflict of interest.
